# Can threatened species adapt in a restored habitat? No expected evolutionary response in lay date for the New Zealand hihi

**DOI:** 10.1111/eva.12727

**Published:** 2018-12-12

**Authors:** Pierre de Villemereuil, Alexis Rutschmann, John G. Ewen, Anna W. Santure, Patricia Brekke

**Affiliations:** ^1^ School of Biological Sciences University of Auckland Auckland New Zealand; ^2^ Institute of Zoology Zoological Society of London London UK

**Keywords:** conservation biology, laying date, *Notiomystis cincta*, phenology, quantitative genetics

## Abstract

Many bird species have been observed shifting their laying date to earlier in the year in response to climate change. However, the vast majority of these studies were performed on non‐threatened species, less impacted by reduced genetic diversity (which is expected to limit evolutionary response) as a consequence of genetic bottlenecks, drift and population isolation. Here, we study the relationship between lay date and fitness, as well as its genetic basis, to understand the evolutionary constraints on phenology faced by threatened species using a recently reintroduced population of the endangered New Zealand passerine, the hihi (*Notiomystis cincta*). A large discrepancy between the optimal laying date and the mode of laying date creates a strong selection differential of −11.24. The impact of this discrepancy on fitness is principally mediated through survival of offspring from hatchling to fledgling. This discrepancy does not seem to arise from a difference in female quality or a trade‐off with lifetime breeding success. We find that start of breeding season depends on female age and average temperature prior to the breeding season. Laying date is not found to be significantly heritable. Overall, our research suggests that this discrepancy is a burden on hihi fitness, which will not be resolved through evolution or phenotypic plasticity. More generally, these results show that threatened species introduced to restored habitats might lack adaptive potential and plasticity to adjust their phenology to their new environment. This constraint is also likely to limit their ability to face future challenges, including climate change.

## 1 INTRODUCTION

Phenology is the study of the timing of life‐history events for individuals, populations and species. Phenological traits, such as the date on which individuals begin to breed, are among the traits most closely linked to individual fitness (Chuine, 2010). In theory, these traits (or, rather, their plastic component) allow individuals to synchronize their life‐history decisions with the time‐related cycles of their environment (e.g., seasonal variation). As such, phenological traits are tightly linked to climatic variation and are involved in biological responses to climate change (Parmesan & Yohe, 2003). Because of their strong links to fitness, phenological traits are one of the possible means for species to adapt to climate change and therefore are of considerable importance for conservation biology (Bellard, Bertelsmeier, Leadley, Thuiller, & Courchamp, 2012).

In birds, the laying date of many species has been shown to be getting earlier in recent decades (Both et al., 2004; Crick, Dudley, Glue, & Thomson, 1997; Parmesan & Yohe, 2003), most likely in response to environmental variation (Both et al., 2004; Visser, Both, & Lambrechts, 2004). Phenological response to climate change is expected to be driven by two mechanisms: (a) phenological traits can be plastic and change at a rapid pace as climatic conditions are changing or (b) they can be under strong selection, due to their influence on fitness in the context of climatic variation, resulting in evolutionary change over time if the trait is heritable. Deciphering the relative importance of both mechanisms is a difficult problem as it requires the genetic basis of phenological traits to be determined (Gienapp, Teplitsky, Alho, Mills, & Merilä, 2008). Although laying date has been found to be heritable and under selection in a few species (Gienapp, Postma, & Visser, 2006; Merilä & Sheldon, 2000; Sheldon, Kruuk, & Merilä, 2003), the expected response to selection may be too small to be detected (Gienapp et al., 2006), or laying date shifts may be constrained by trade‐offs with other traits (Brown & Brown, 1999). As a result and given other empirical results showing that laying date seems to largely plastic (Charmantier et al., 2008; Gienapp et al., 2008), the hypothesis of phenotypic plasticity is often favoured. Phenotypic plasticity is however not always adaptive (Ghalambor, McKay, Carroll, & Reznick, 2007), and the ability of bird species to efficiently adjust their phenology to climatic change through a plastic response will essentially depend on the environmental cue used (Chevin & Lande, 2015; Visser et al., 2004) and how well it predicts the optimal phenological timing. Indeed, depending on the relevance of the signal used to plastically alter the laying date, the mechanism could (a) help better track the optimum of environmental resources, (b) be totally independent of environmental resources or (c) even trigger a response in the wrong direction. For example, change in photoperiod is a possible cue for the beginning of spring but, being independent from climate, it is not expected to trigger adaptive phenotypic plasticity to an earlier spring onset as a result of climate change. One of the most efficient environmental cues related to climate change is temperature, which seems to be the cue used by the most studied passerine species to determine lay date (Caro, Schaper, Hut, Ball, & Visser, 2013; Crick et al., 1997; Phillimore, Leech, Pearce‐Higgins, & Hadfield, 2016). Most of the evolutionary research presented above has however been performed on non‐threatened, mostly European, birds (but see Teplitsky, Mills, Yarrall, & Merilä, 2010, for a study on a non‐threatened New Zealand endemic bird). As a consequence, little is known about the potential for an evolutionary or plastic response of threatened birds, or more generally for any endangered species, typically existing in degraded habitat or in small and isolated populations with potentially limited genetic diversity.

The hihi (stitchbird, *Notiomystis cincta*) is a New Zealand endemic and threatened passerine bird. Once spread over most of the North Island, hihi are now naturally occurring only on Hauturu‐o‐Toi (Little Barrier Island; 36°12′S, 175°05′E) and in six reintroduced populations spread across their former range. Natural dispersal is not possible for the hihi between these seven populations (due to long distances between them and the inability of the hihi to survive for a long enough period outside pest‐free sanctuaries), and no occurrence of natural re‐colonization has been observed since the hihi population collapse across the North Island in the late 1800s. As a result, a response of the species to climate change will either depend on adaptation to these changes (through evolutionary processes or phenotypic plasticity), human intervention such as translocation to more suitable habitats (Chauvenet, Ewen, Armstrong, & Pettorelli, 2013) or, more generally, appropriate changes in management strategies. Additionally, some of the environments within which hihi were reintroduced are currently recovering from heavy degradation (e.g., almost complete land clearing), by contrast with the only native population consisting predominantly of mature forest (Makan, Castro, Robertson, Joy, & Low, 2014). This means that introduced populations are not expected to be at evolutionary equilibrium with these growing, immature forests, possibly resulting in a strong mismatch between their phenology and resource availability (Gienapp et al., 2008), reducing population fitness. If this is the case, the currently increasing additional pressure from climate change might pose a threat to their conservation in these environments.

In this study, we investigate the evolutionary aspects of laying date in the hihi using a particularly extensive data set from a threatened bird species, which includes data on nest lay date, fledgling recruitment, survival and individual fitness. We assess in particular whether the heritability, selection and plasticity typical of laying date described previously in non‐threatened bird species apply to this threatened species. We use long‐term data on a pedigreed wild population of hihi to explore variation in laying date, its genetic basis and the selective pressure upon this variation, in order to determine whether (a) lay date is under stabilizing selection with an optimum of fitness, and if so, whether the observed lay date matches this fitness optimum, and (b) if a discrepancy with this optimum occurs, whether this trait has enough adaptive potential (i.e., trait heritability) to evolve in response to selection.

## 2 MATERIAL AND METHODS

### 2.1 Data sampling

Here, we focus on a restored mammalian pest‐free island, Tiritiri Matangi (36°36′S, 174°53′E), in which individuals were released from the island Hauturu‐o‐Toi in August 1995, August 1996 and March 2010. The population has been closely monitored since 1995 with all birds individually identifiable with leg bands (almost exclusively applied as nestlings) and with most nesting attempts known. No natural immigration to or emigration from the island has been observed. Hihi feed on a mix of fruits, nectar and small invertebrates (Castro, Minot, & Alley, 1994), but are also provided with supplementary food (20% by mass sugar water). The population grew since its establishment in 1995 to an artificially managed carrying capacity (ca. 150) reached in 2005–2006, due to semi‐regular harvests (every ~2–3 years) of fledglings to source individuals for new translocation events (Armstrong & Ewen, 2013). During harvests, at most 20% of fledglings are taken at random from the population, with translocations taking place between March and May, the austral autumn. The majority of juvenile mortality occurs in the first 8 weeks after fledging (Low & Pärt, 2009), so most individuals harvested are likely to be fit enough to recruit and by this point most selection would have already taken place (Armstrong & Ewen, 2013).

Hihi usually reproduce in their first year, during the austral spring and summer (September to February, Castro et al., 1994). Females lay clutches ranging from three to five eggs, at 25‐hr intervals and can produce multiple clutches within a season although normally only one or two are successful. Despite males providing around 30% of the care during rearing (Ewen & Armstrong, 2000; Low, Joy, & Makan, 2006), extrapair paternity in this species is widespread. Around 60% of chicks within a brood are sired by extrapair males (Brekke, Cassey, Ariani, & Ewen, 2013). Hihi usually lay eggs in natural tree cavities, but given that the forest on Tiritiri Matangi is immature, such cavities are scarce. Instead, nest boxes are provided for hihi across the island, within which the vast majority of breeding events occur. As nest boxes and food are provided ad libitum by management, and the population is subject to periodic removal of fledglings for translocation to other populations, there is very little evidence of density‐dependent survival in the population (Armstrong & Ewen, 2013). During each nesting attempt, we record (a) the identity of the (social) sire and the dam; (b) lay, hatch and fledge dates; and (c) the corresponding numbers of eggs/chicks at each stage. Surviving fledglings are measured, banded and blood‐sampled. The intense monitoring (since 1995), combined with a microsatellite genotyping effort started in 2004 (Brekke, Dawson, Horsburgh, & Ewen, 2009), allowed us to reconstruct a long‐term pedigree of the Tiritiri Matangi population, while accounting for extrapair paternity (Brekke et al., 2012).

We used temperature data from 1995 to 2010, downloaded from the New Zealand National Climate Database (https://cliflo.niwa.co.nz/), to assess the relationship between laying date and temperature. The years cover a period over which a weather station from New Zealand's National Climate was present on the island. As a proxy for the yearly environmental cue, we used the average maximal temperature for 50 days prior to the grand mean of the start of breeding season (see below) across years, as sliding window approaches often identify this as the most predictive period (McLean, Lawson, Leech, & van de Pol, 2016).

### 2.2 Phenotypic information

This study is based on the breeding seasons spanning from 1997/1998 to 2013/2014. Lay date was derived directly from the data, except for the first season 1997/1998 where the data were missing. Instead, it was computed as being 17 days before the recorded hatch date (regression of laying date on hatch date, *N*_obs_ = 1,207, *a* = −17.07 days, *b* = 1.0, *R* ^2^ = 0.998). Dates require a point of reference, so for the analyses of this article, the laying date was defined as the number of days since the 1st of September of the year corresponding to the breeding season. Attempts at breeding by females were numbered with an increasing clutch number, independently of the success of the clutches. In the following, “start of breeding season” corresponds to the laying date of the first of these successive clutches or of the sole clutch if only one has been recorded. The fitness of the breeding female was computed as the number of offspring recruited as breeders in the following generations (using both the social and genetic pedigrees, see below). The age of females was taken as the number of years between the focal season and the season of the birth of the female. Because hihi have a limited growth after fledging (Low, 2006), we used the tarsus length measured when individuals are banded (as 21‐day‐old nestlings) as a measure of the individual adult size. The total data set consisted of 1,369 breeding events (855 whole‐season events) for 330 females, spanning 16 years.

### 2.3 Pedigree reconstruction

The social pedigree was reconstructed based on the colour band information of the sire and dam observed at each nest box. Due the high level of extrapair paternity, we used a panel of microsatellite markers (Brekke et al., 2009) and the COLONY software (Wang, 2004, 2012; Wang & Santure, 2009) to reconstruct the paternity. For the microsatellite markers, genomic DNA was extracted for samples from blood and tissue samples during 2007–2012 using the Promega Wizard^®^ SV genomic DNA purification system (PROMEGA) following the manufacturer's instructions. Samples collected prior to 2007 were extracted using the ammonium acetate precipitation method. All samples were genotyped at 18 microsatellite loci, 15 were species‐specific and three were designed for other passerine species (see Brekke et al., 2009; for extraction and genotyping details). Sex was identified using two fluorescently labelled primers (Z002A and Z037B; Dawson et al., 2007) and where possible in combination with adult plumage morphology. For COLONY, in brief, all behavioural maternities of clutches were assumed correct and specified as such. For clutches where behavioural observation of maternity was not available, only maternal sibship was specified but not maternal ID. Candidate fathers included any known male alive in the pre‐breeding September census and post‐breeding February census (not born that season) and also all identified territorial males. The probability of the true parents being in the candidate lists was set at 0.90 for females and 0.80 for males as a number of males do not hold territories (≃30%) and may still gain extrapair copulations (Brekke, Ewen, Clucas, & Santure, 2015). These probabilities reflect a very high probability of recapture of the individuals on the island (Chauvenet et al., 2013). Both sexes were defined as polygamous and allele frequencies and genotyping error rates were provided as input (between 0 and 0.012 depending on the locus, estimated from repeat genotyping of ≃10% of samples). Because blood sampling was only initiated in the 2003/2004 breeding season, information relating to the genetic sire of individuals born previously is missing. For these individuals, we considered the information as missing, rather than using the social sire (hereafter termed the “full pedigree”). The maximal depth of this pedigree was 13 generations with an average of 6.69. We also constructed a pedigree restricted to the years where the genetic information is available (from 2003 to 2014, termed the “subset pedigree”). This pedigree had a maximum depth of 10 with an average of 4.2 generations. We computed individual inbreeding coefficients using the whole pedigree record, but only for individuals with known parents and grandparents. Inbreeding coefficients for individuals with at least one unknown parent or grandparent were considered as missing values.

### 2.4 Statistical analysis

#### 2.4.1 Start of breeding season, reclutching and female survival

To study the influence of female quality on variation in the start of the breeding season, probability of reclutching and female survival, we used three different proxies for female quality: age, size and inbreeding.

First, to study the variation in the start of breeding season, we used a mixed modelling approach where age, size and inbreeding were fitted as fixed effects and the identity of the female and year were fitted as random effects, to model random between‐individual and between‐year variation. Because the relationship with age seemed nonlinear, we evaluated the fit of a linear model, a quadratic model, a broken lines model with a break at age 2, a broken lines model with breaks at ages 2 and 6 and a broken lines model with a different slope for each transition in age. Once the best model was determined, we tested the continuous effect of time (year, standardized, i.e., mean‐centred and scaled to a variance of 1) to detect variation in the start of breeding season over the study period. This effect was tested with and without year as a random effect (to remove covariation when year is fitted as both fixed and random and therefore increase power to detect a linear trend).

Second, we studied the probability of reclutching (having more than one clutch) by fitting a binomial model using start of breeding season and female quality proxies (age, size and inbreeding) as fixed effects with female identity and year as random effects. For the effect of age, we tested a linear effect, a difference between 1‐year‐old and older females and a difference between 1‐year‐old, middle‐aged (from 2 to 6 years) and older females (above 6 years).

Third, we studied whether female survival to the subsequent year depended on when she started breeding, the number of clutches per year and her quality in binomial mixed models with year and female identity as random effects.

Fourth, we studied the existence of phenotypic plasticity with temperature, using the average temperature cue described above. To do so, we fitted an individual model of the start of breeding season using the best model from the first analysis above and tested whether the inclusion of the temperature cue as a new variable was significant in the model.

To help convergence of the algorithms, continuous variables (except inbreeding) were standardized, that is, mean‐centred and scaled to a variance of 1. The models were fitted in R (R Core Team, 2017) with the lme4 package (Bates, Mächler, Bolker, & Walker, 2015) and were compared using Akaike's information criterion (AIC, Akaike, 1981; Burnham & Anderson, 2002). The AIC was computed using maximum likelihood while the estimates provided throughout the article were computed using restricted maximum likelihood. The significance of variables for the model was tested by comparing AIC as follows: Variables were evaluated separately against a null model. Variables that were not significant on their own were discarded. Variables that were significant on their own were all included in a full model and compared to models with each variable dropped one by one. The best model was chosen, and variables were tested (dropped) again until a stable state was reached. The best inferential model was considered as the most parsimonious one with a contrast to the best predictive model ΔAIC < 2. Within the best model, the significance of each parameter (departure from zero) was tested using the lmerTest R package.

Because including inbreeding was generating a predictor with a lot of missing values (hence a greatly reduced subset of the data), we tested this variable separately by comparing a null model, a model with inbreeding and the best model with or without inbreeding.

#### 2.4.2 Heritability of laying date

To estimate the heritability of female laying date, we used the R package MCMCglmm (Hadfield, 2010). We conducted the analysis either using all data available (i.e., full pedigree) or restricting to years where molecular data were available to reconstruct the pedigree (i.e., subset pedigree). Although the distribution of laying date is skewed, it was analysed as a Gaussian trait as using the clutch number within a year as a fixed effect was an efficient way to account for the skewness. In addition to the additive genetic effect, the female identity (permanent environment effect), the social male identity and the year were fitted as random effects. The phenotypic variance was computed as the sum of all random effect variances, the residual variance and the variance arising from fixed effects, following de Villemereuil, Morrissey, Nakagawa, and Schielzeth (2018). The prior for these random effect variances used the parameter extension implemented in MCMCglmm with parameters *V* = 1, nu = 1, alpha·mu=0 and alpha·*V* = 1,000. The prior parameters for the residual variance were set to *V* = 1 and nu = 0.02. Clutch number was included as a fixed effect, and its significance was tested using the pMCMC value yielded by MCMCglmm. The models were run for 500,000 iterations with a thinning interval of 10, after a burn‐in of 3,000. These parameters were chosen to ensure a MCMC effective sample size above 8,000 for all parameters. Convergence for all parameters was checked graphically and by using the Heidelberger and Welch (1981) test as implemented in the coda R package (Plummer, Best, Cowles, & Vines, 2006). The heritability of laying date was computed as the ratio between the additive genetic variance and the sum of all variance components in the model excluding the between‐year variance (i.e., phenotypic variance within years).

#### 2.4.3 Power analysis

To evaluate the capacity of our data to estimate low levels of heritability, we performed a power analysis. We used our exact data structure (pedigree, number of individuals and structure of multiple measurements), but simulated a new phenotypic trait. We simulated breeding values according to our pedigree using the MCMCglmm rbv() function, as well as all the other random effects fitted in the above model (permanent environment, mate and year effects, all with the same variance). Variance components were set so that the total variance was comparable to our laying date data set and the resulting expected heritability would be 0.1, as estimates below this threshold would be typically considered as small. As a comparison, using the meta‐analysis data set from Mittell, Nakagawa, and Hadfield (2015), we found that heritabilities reported for passerine laying date typically range from 0.09 to 0.265 with an average of 0.14 (*n* = 8). We replicated this simulation 100 times and analysed each simulated data set using MCMCglmm to estimate heritability. We computed the posterior mean, median and 95% credible interval for each replicate.

#### 2.4.4 Optimum inference

For six fitness‐related traits ((a) fitness as defined previously as the number of offspring recruited as breeders in the following generations, (b) the number of eggs laid, (c–e) survival through the three different juvenile stages egg—hatchling, hatchling—fledgling, fledgling—recruit, and (f) survival from egg—recruit), the presence of an optimum was first tested using a generalized linear mixed model (GLMM) including a first‐ and second‐order effect of laying date (as well as various other confounding effects such as the size, inbreeding and age of the female and the clutch number of the season). For each fitness‐related trait, we compared the model including the second‐order effect with a model without using AIC: If the difference in AIC was larger than 2, indicating support for an optimal value, we proceeded by using the following model to infer the value of the optimum. The main reason for using the model below rather the GLMM is that the optimum, which is the parameter of interest here, is a compound function of the first‐ and second‐order parameters of the GLMM. This makes the computation of uncertainty measures (such as confidence/credible intervals) and the inclusion of a year‐to‐year variation of the optimum complex. In order to infer the optimum of these fitness‐related traits depending on lay date, we considered a model (akin to the model described in equation 1 of Chevin, Visser, & Tufto, 2015) where the latent response *Z* was evaluated as a Gaussian curve depending on the (mean‐centred and scaled to a variance of 1 across all years) laying date *x*_*i*_ (here thus a covariate of the model) for each individual breeding record *i* during year *j*(*i*), the optimal date *λ*_*j*(*i*)_ depending on the year *j*(*i*), the dispersion coefficient around the optimum *σ* and a scaling factor *A*.

(1) $$Z_{i}\;=\;A\exp\left[-{\left(\frac{x_{i}-\lambda_{j(i)}}{\sigma }\right)}^{2}\right].$$

Note that this estimates one optimum per year *j*. The optimal date was modelled as depending on the year as a random effect:

(2) $$\begin{array}{cc} \lambda_{j} & \;=\;\mu +u_{j},\\ u_{j} & \;∼N_{\left[-2,2\right]}\left(0,\sigma_{U}^{2}\right), \end{array}$$

where *μ* is an across‐year intercept, *u*_*j*_ is a year‐dependent random effect with variance $\sigma_{U}^{2}$ and $N_{\left[-2,2\right]}$ is a normal distribution truncated between [−2, 2]. The realized response *Y* (i.e., the observed data) is then modelled according to either a Poisson (for fitness) or binomial (for survival) error distribution, hereafter denoted as D:

(3) $$Y_{i}\;=\;D\left(Z_{i}\right).$$

Prior distributions for the total model were as follow:

(4) $$\begin{array}{cc} \mu & ∼U(-2,2),\\ \sigma & ∼U(0,100),\\ A & ∼U(0,A_{\text{max}}),\\ \frac{1}{\sigma_{U}^{2}} & ∼\Gamma (0.001,0.001) \end{array}$$

where U stands for a uniform distribution and Γ stands for the Gamma distribution. The upper bound of the prior for the dispersion parameter *σ* was set to a high value so that the model was able to yield a flat line, in case the most likely model is one without an optimum. The upper bound of the scale parameter *A*, here denoted *A*_max_, was equal to 5 for fitness and 1 for survival. The relatively narrow distribution for *λ*_*j*_ = *μ* + *u*_*j*_ was chosen to ensure that the fitted optimum belongs to the realm of possible laying date with boundaries −2 and 2 roughly corresponding to the 2% and 98% quantiles of the scaled laying date variable.

For each fitness‐related trait, the model above was implemented in JAGS (Plummer, 2003). The total model was run in eight chains for 50,000 iterations with a thinning interval of 10 after a burn‐in of 3,000. These parameters were chosen to ensure a total effective sample size of the MCMC above 10,000. The convergence was checked by comparing the eight chains using the potential scale reduction factor of Gelman and Rubin (1992) as implemented in the coda R package (Plummer et al., 2006). All parameters had a factor equal to 1, meaning that the same convergent state was reached by the different chains. As lifetime reproductive success tend to be zero‐inflated, we performed posterior predictive checks (Gelman, Meng, & Stern, 1996; Rubin, 1984): We simulated new data according to the model and posterior distributions and compared their distribution to the distribution of our data. A model including an individual random effect (based on female ID) was considered but is not included here as it took longer to run with no substantial difference in the results. Finally, we fitted a model using information regarding the temperature cue for each year (termed *θ*_*j*_), to check whether this would improve the fit of the model and hence the inference of the optima. This model is the same above, with an additional slope *B* between the optimum and temperature:

(5) $$\lambda_{j}\;=\;\mu +B\theta_{j}+u_{j}.$$

The prior of the slope was defined as a vague normal distribution with mean 0 and variance 10^6^. The significance of the slope was tested as twice the proportion of iterations of a different sign than the posterior median.

Optima were compared to the mode (computed as the optimum of the density distribution of laying date), rather than the mean, because the distribution of laying date is skewed towards later dates which would influence the mean but not the mode. We do not comment on year‐to‐year estimated variation in optima, as low sample sizes led to estimates that appeared too unreliable for some years to be able to robustly infer a temporal trend. The selection differential of laying date was computed as the covariance between this trait and relative fitness (Robertson, 1966, 1968), and standardized selection gradients were computed using Lande and Arnold (1983)'s framework. Standard errors were obtained from a non‐parametric bootstrap for the selection differential and from the standard errors of the linear model estimates for the gradients.

#### 2.4.5 Survival analysis and laying date

We studied the survival between different stages of development (egg, hatchling, fledgling, recruit; with recruit being defined as breeding for at least 1 year) according to laying date (as a quadratic effect), clutch number and female quality (age, size and/or inbreeding). To keep the number of models tested low, only one model for age was tested: young (1‐year‐old)/middle (2‐ to 6‐year‐old)/old (>6‐year‐old). This was done using a binomial mixed model with year and female identity as random effects. As above, the models were fitted in R with the lme4 package and compared using AIC using the procedure described previously. To improve model fit, laying date and size were standardized (mean‐centred and scaled to a variance of 1). When a quadratic effect of laying date was significant, we also used the optimum model described above to estimate the optimum of survival according to laying date.

## 3 RESULTS

### 3.1 Start of breeding season

**Figure 1:**
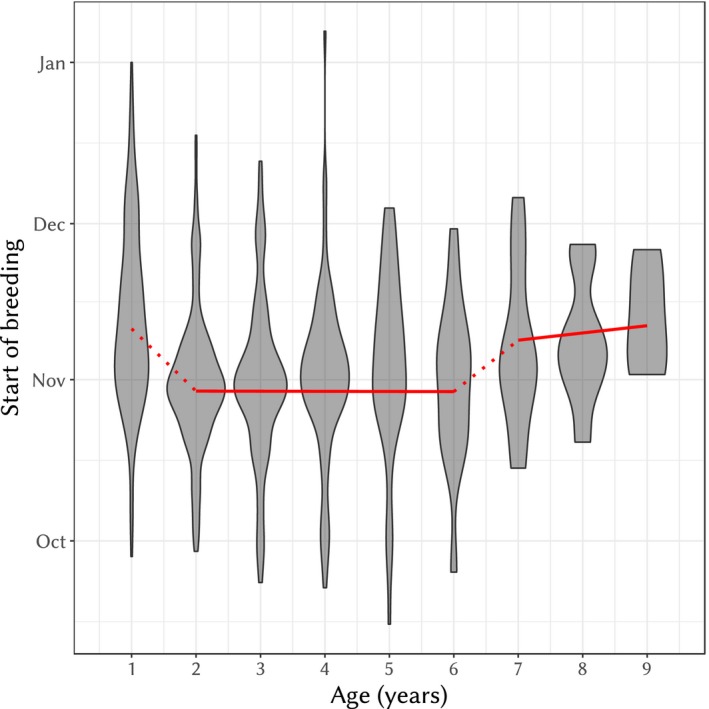
Violin plot of the start of breeding season according to the age of the female. The grey‐filled area depicts the density of start date values conditional on age (i.e., width is not comparable between different ages, to improve readability). The solid red line is the prediction from the best model (i.e., the broken lines model with breaks at ages 2 and 6). The dotted parts are the discontinuities in the model

The start of breeding season depended on both age and size of the female. The best model to predict the influence of the age of female on the start of breeding season was the broken lines model with breaks at age 1 and 6 and size as covariates (see Supporting Information Table S1). The predicted trend in this best model (red line in Figure 1) shows that females in their first year lay eggs later (effect ± *SE* = 12.5 ± 1.38 days, *t*_506_ = 9.06, *p* < 10^−15^). This is also the case for old females (i.e., of age over 6) with a significantly positive slope (slope ± *SE* = 1.33 ± 0.426 days/year, *t*_486_ = 3.13, *p* = 0.00184, see also Figure 1). Between the ages of 2 and 6, however, the start of breeding season is earlier and does not significantly depend on age (slope ± *SE* = 0.228 ± 0.476 days/year, *t*_639_ = 0.480, *p* = 0.632, see also Figure 1). The start of breeding season was further negatively dependent on the tarsus size of the female (slope ± *SE* = −1.70 ± 0.749 days/mm, *t*_245_ = −2.28, *p* = 0.0237), although it had a relatively small effect in relation to female age. Inbreeding was not significant (see Supporting Information Table S1).

**Figure 2:**
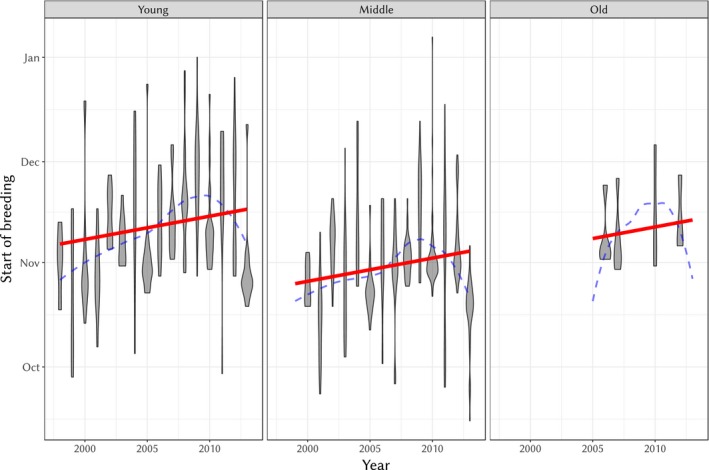
Violin plot of the start of breeding season according to years for each age class (young: 1‐year‐old, middle: between ages 2 and 6, old: older than 6). The grey‐filled area depicts the density of start date values for each year (note that width is not comparable between different years). The solid red line is the prediction from the best model (i.e., the broken lines model with breaks at ages 2 and 6 with tarsus size as a covariate, not shown here). The blue dashed line is a LOESS estimate (from the ggplot2 R package, Wickham, 2009) of the relationship between start of breeding and year

There was weak evidence for a temporal trend in the start of breeding season. Using the best model above and including years as both a fixed (continuous) effect while keeping it as a random (categorical) effect, thus accounting for both a linear trend and random among‐year variation, did not result in a significant improvement of the model (with year as fixed effect, AIC = 5,631.0; without, AIC = 5,632.6). When year as a random effect was removed from the model, the continuous effect for year became significant (with year as fixed effect, AIC = 5,845.2.0; without, AIC = 5,847.5), although the overall fit was worse than with the random effect alone. The slope in the latter model was positive and relatively strong (slope ± *SE* = 2.62 ± 0.68 days/year, *t*_291_ = −3.85, *p* = 0.000146, see Figure 2). Any relationship between start of breeding season and time is thus most likely masked by random year‐to‐year variation on the scale of the study (see LOESS estimate, blue dashed line in Figure 2), but these results show that should it be changing over time, it would be towards later, rather than earlier dates.

**Figure 3:**
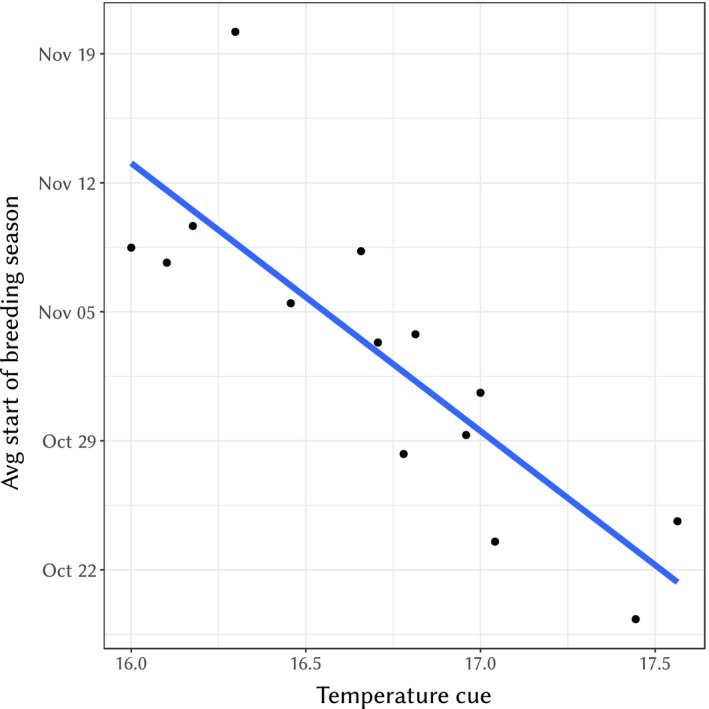
Average start of breeding season (over all breeding females) each year against the temperature cue (average temperature 50 days prior to the grand mean of start of breeding season across the years). The blue line is only illustrative and is based on a simple linear modelling of the data points without other fixed and random effects, see main text for a more refined slope estimate

In contrast, the relationship between start of breeding season and our temperature cue was well supported. Adding the temperature variable to our best model of start of breeding season resulted in a significantly better fit (AIC = 708.54, compared to AIC = 724.61 without the temperature cue). Lower temperatures 50 days prior to the average start of breeding season lead to a delayed start of breeding (slope ± *SE* = −18.28 ± 3.24 days/°C, *t*_14.1_ = −5.65, *p* = 5.86 × 10^−5^, see Figure 3). No linear trend (e.g., average increase in temperature over time) could be detected for the temperature cue over the years (slope ± *SE* = −0.036 ± 0.031°C/year, *t*_12_ = 1.41, *p* = 0.275).

### 3.2 Probability of reclutch

**Figure 4:**
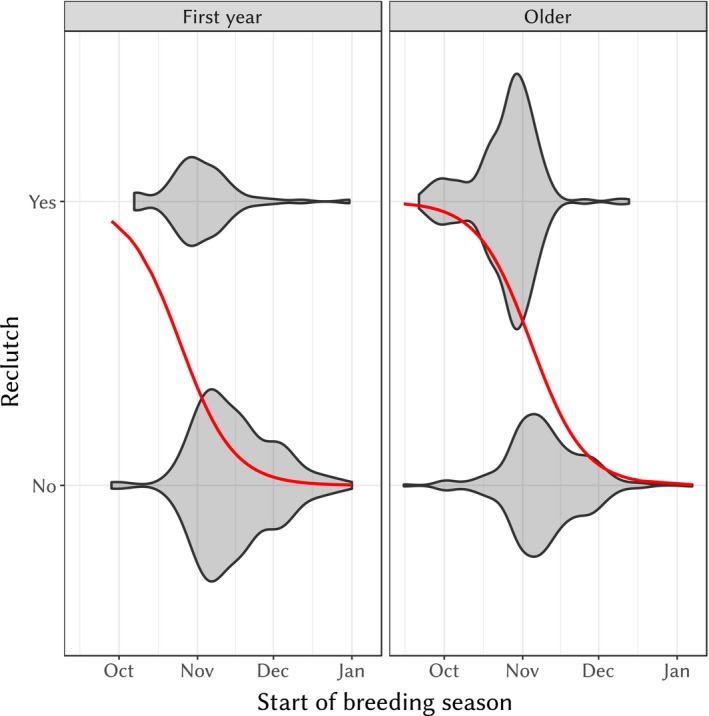
Violin plot of “reclutching” (having at least one other clutch after the first clutch) for females against the start of breeding season for young females (first year) and older ones (second years and older). The red solid line is the predicted probability of reclutching

Older and earlier breeding females tended to reclutch more often than younger and later breeding females. The best model for the probability of reclutch included start of breeding season and first‐year/older females effects (see Supporting Information Table S2). First‐year females tended to reclutch less than older females (effect ± *SE* = 1.10 ± 0.220, *z* = 4.97, *p* = 6.69 × 10^−7^) and the effect of start of breeding season was negative (slope ± *SE* = −1.53 ± 0.165 day^−1^, *z* = −9.32, *p* < 10^−15^, see also Figure 4). Size and inbreeding did not significantly influence the probability of laying more than one nest (see Supporting Information Table S2). Despite the relationship between probability of reclutch and start of breeding season, the total number of fledglings over a year for a female did not depend on the number of clutches when the start of breeding season was included in the model (generalized mixed model with Poisson distribution including the effect of both variables: AIC = 2,844.3, with only start of breeding season: AIC = 2,844.7 or with only the clutch number, AIC = 2,949.0).

### 3.3 Female survival to the next season

Surival was associated with a larger number of clutches, but not with the start of breeding season. The best model for female survival to the next season included age as a continuous effect and the number of clutches during the breeding season (see Supporting Information Table S3 and Figure S1a). Thus survival was not significantly associated with the start of breeding season (see Supporting Information Figure S1b). It was however negatively associated with age (slope ± *SE* = −0.83 ± 0.318 year^−1^, *z* = −2.61, *p* = 0.00906) and positively associated with the number of clutches during the breeding season (slope ± *SE* = 0.734 ± 0.230, *z* = 3.18, *p* = 0.00145).

### 3.4 Heritability of laying date

The estimated heritabilities of laying date were very low, with a lower bound of the credible interval near zero (Table 1), despite statistical support away from the prior, shown by the agreement between the posterior mode and median. This low heritability resulted from a low additive genetic variance, also with a lower bound of the credible interval near zero. The variance estimates for laying date were comparable between the two analyses using all years available (full pedigree) or only years with genotypic information (subset pedigree, see Table 1). The heritability computed from the mother–daughter regression was also very low (Table 1) and not significant (*t*_241_ = 0.053, *p* = 0.958). The variance of the permanent environment effect (*V*_PE_) was estimated as being larger than the additive genetic variance (*V*_A_), but with a similar order of magnitude. In combination, these two effects lead to a small repeatability (although supported as being away from zero in the model) of the laying date (*r* ^2^ in Table 1). Our power analysis, which simulated a heritability of 0.1 (Supporting Information Figure S2) shows that the pedigree had sufficient power to detect a moderate heritability, and further that the probability of obtaining a heritability estimate as low as or lower than ours is minimal, with 75% (94%) of the replicates with posterior mode (median) higher than those calculated from our true data set. Combining this power analysis with our upper credible interval bound, it is likely that the true heritability of laying date of the hihi is lower than 0.1. Clutch number was a significant effect in our models (pMCMC < 10^−5^).

**Table 1:** Variance decomposition, heritability (*h* ^2^) and repeatability (*r* ^2^) estimates for laying date from the animal model using the whole data set (full animal model, years from 1997 to 2014) or the subsample using only years with genotypic information (subset animal model, years from 2003 to 2014)

Parameter	Full animal model	Subset animal model	Mother–daughter regression
Mean	83.2 (84) [79–89]	82.5 (83) [77–88]	—
*V*_F_	572 (576) [539–611]	569 (569) [534–607]	—
*V*_Year_	58.3 (76) [30–152]	77.4 (90) [37–188]	—
*V*_Mate_	14.6 (17) [5.9–31]	20 (18) [5.9–32]	—
*V*_PE_	46.1 (43) [11–76]	41.6 (42) [14–72]	—
*V*_A_	27 (31) [1.7E‐5 to 67]	19.7 (21) [1.2E‐6 to 51]	—
*V*_R_	161 (162) [146–178]	164 (164) [148–181]	—
*V*_P_	832 (831) [784–881]	818 (818) [771–867]	—
*h* ^2^	0.0322 (0.037) [2.1E‐8 to 0.079]	0.0246 (0.026) [1.4E‐9 to 0.061]	0.0103 ± 0.19
*r* ^2^	0.0931 (0.091) [0.065–0.12]	0.0811 (0.079) [0.054–0.11]	—

Notes

Point estimates are given using the following format: posterior mode (posterior median) [95% credible interval]. The heritability estimate and corresponding confidence interval from a mother–daughter regression (using all years) is given in the third line. Units for the mean are days and for variances are days^2^.

*V*_F_: variance arising from fixed effects; *V*_Year_: between‐year variance; *V*_Mate_: between social sire mate variance; *V*_PE_: permanent environment variance; *V*_A_: additive genetic variance; *V*_R_: residual variance; *V*_P_: total phenotypic variance (excluding *V*_Year_).

### 3.5 Optimum of fitness and initial investment according to laying date

#### 3.5.1 Optimum of fitness

**Figure 5:**
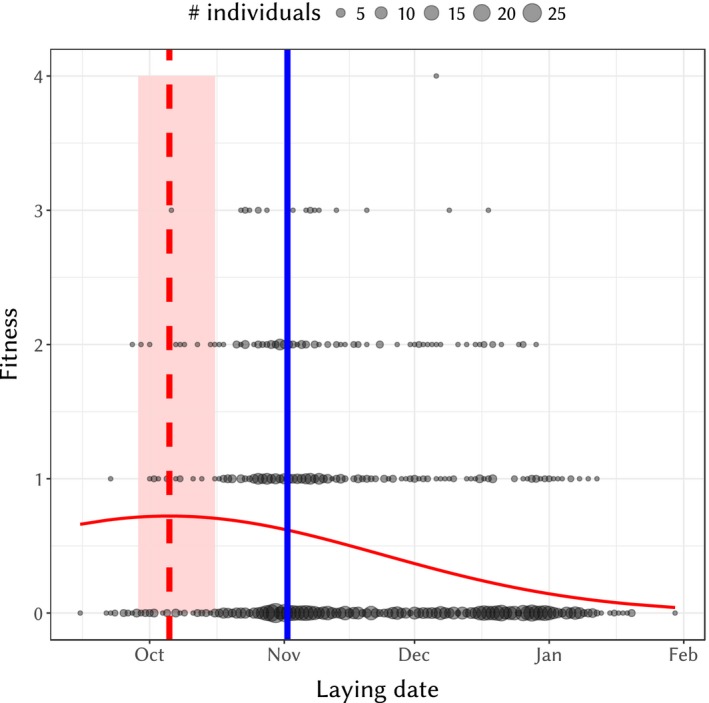
Fitness against laying date. Circle sizes are proportional to the number of individuals sharing the same fitness value and laying date. The red curve is the fitted model, vertical red dashed line is the optimum and the light red area depicts the 95% credible interval of the optimum. The vertical solid blue line is the mode of laying date. Fitness is defined as the number of offspring recruited as breeders in the following generations

There was a fitness optimum for laying date, with a significant quadratic effect of laying date on fitness (number of recruited offspring *per* brood) when fitting a generalized linear mixed model with a Poisson distribution and year as a random effect (AIC_laying date_ = 1,966.2, AIC_null_ = 2,067.6). The selection differential and standardized selection differential were estimated (standard errors within parenthesis) as −11.24 days (1.25) and −0.408 (0.045), respectively. The standardized linear and nonlinear selection gradients were estimated (standard errors within parenthesis) as −0.38 (0.049) and −0.268 (0.098), respectively. Using the model of Equation (1), the overall optimum of fitness was estimated as October 5th, with a 95% credible interval between September 28th and October 16th (Figure 5). The mode of laying date was estimated as November 1st, hence outside of the 95% credible interval for the fitness optimum. Our model was a good fit for the fitness distribution, despite a slight enrichment in zero values in the data compared to replicated data in a posterior predictive check (Supporting Information Figure S3). When using only birds of good “quality” a priori best able to target the optimum using two different criteria: (a) females from ages 2 to 6, (see Figure 1) or (b) females that survived to the next year, the effect is still significant (mode outside the 95% credible interval of the optimum, see Supporting Information Figure S4). The discrepancy between the optimum and mode was also still significant when analysing the start of breeding season against the annual fitness (cumulated number of fledglings across breeding events within a year, see Supporting Information Figure S5) Both for the general and fitter populations, the mode of laying date was thus significantly later than the optimum of fitness. Finally, the optimum did not significantly depend on the temperature cue, as the slope of the relationship between the cue and the yearly optima did not differ significantly from zero (slope *B* ± *SE* = −0.098 ± 0.22, pMCMC = 0.747, unstandardized slope = −6.849).

#### 3.5.2 Optimum of initial investment

**Figure 6:**
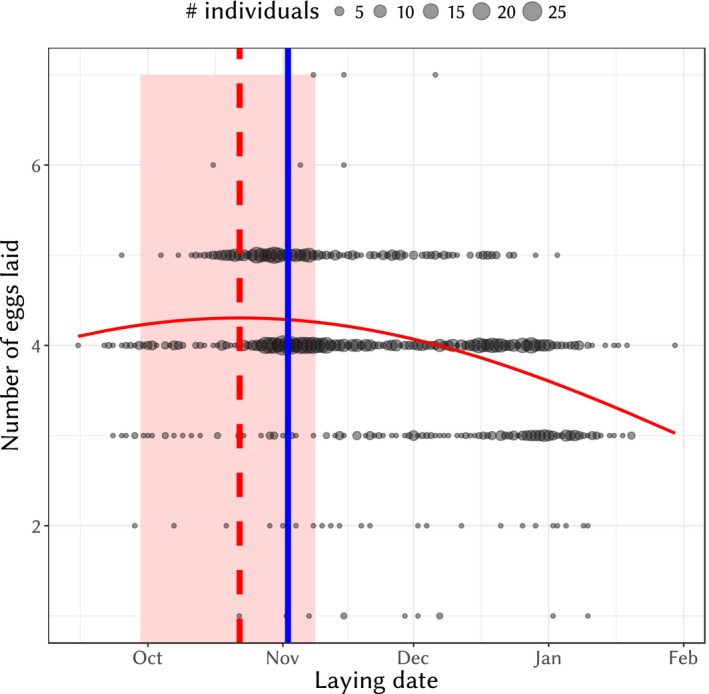
Relationship between number of eggs laid and lay date. Circle sizes are proportional to the number of individuals sharing the same initial investment value and laying date. The red curve is the fitted model, vertical red dashed line is the optimum and the light red area depicts the 95% credible interval of the optimum. The vertical solid blue line is the mode of laying date

The significance of an optimum of the initial investment (number of eggs laid) according to laying date was confirmed using a generalized linear mixed model with a Poisson distribution and year as a random effect (AIC_laying date_ = 4,594.6, AIC_null_ = 4,621.4). Compared to the optimum of fitness, the optimum in initial breeding investment is inferred with more uncertainty, due to the effect of laying date being less strong (Figure 6). The optimum date of laying is inferred as being October 22nd with a 95% credible interval between September 29th and November 8th. It is thus not significantly different from the mode of laying date (November 2nd in this subset of the data).

### 3.6 Survival between juvenile stages

#### 3.6.1 Egg to hatchling

**Figure 7:**
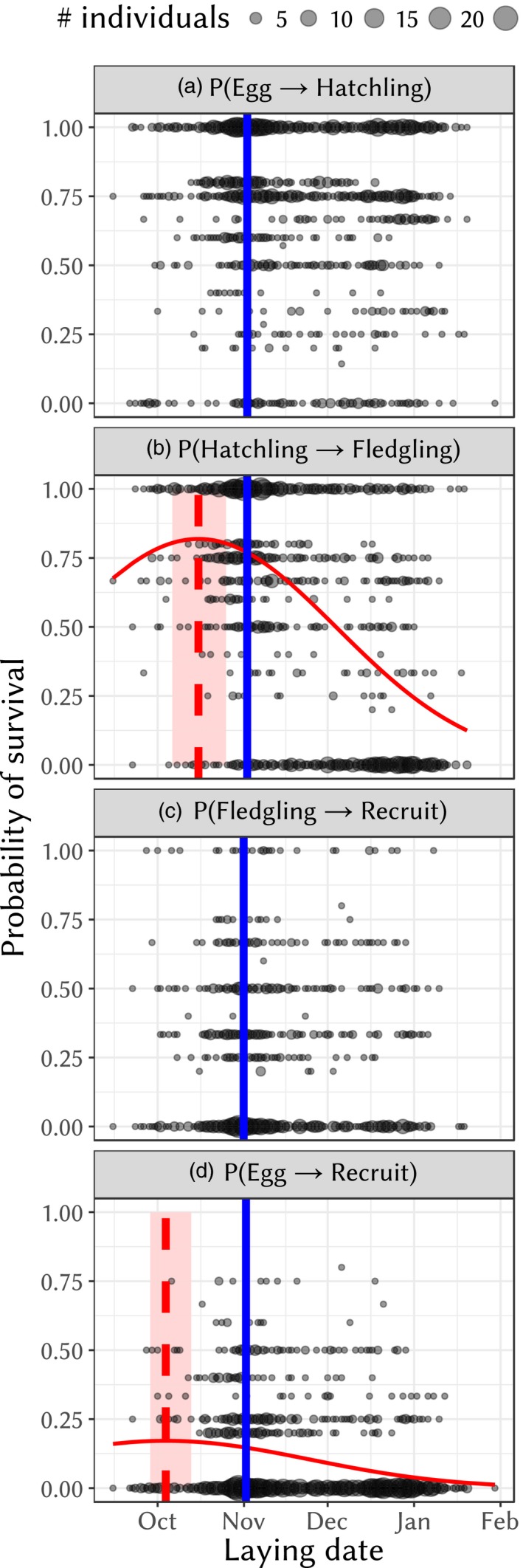
Probability of survival between different development stages (egg, hatchling, fledgling, recruit). Circle sizes are proportional to the number of individuals sharing the same realized value (ratio, for a given female, of the number of offspring alive at the later stage to the number of offspring alive at the earlier stage) and laying date. The red curve is the fitted model, vertical red line is the optimum and the light red area depicts the 95% credible interval of the optimum (figures with no red are those with no optimum inferred). The vertical solid blue line is the mode of laying date for the particular subset of the data corresponding to the graph

There was no significant optimum of survival from egg to hatchling according to laying date. The best model for this variable included age and clutch number as fixed effects (Supporting Information Table S4). When compared to middle‐aged females (of ages 2–6), the probability of hatching was significantly lower for older females (effect ± *SE* = −0.788 ± 0.193, *z* = −4.09, *p* = 4.41 × 10^−5^), but not for females in their first year (effect ± *SE* = −0.102 ± 0.0897, *z* = −1.14, *p* = 0.256). It was negatively associated with clutch number (slope ± *SE* = −0.180 ± 0.06917, *z* = −2.60, *p* = 0.0092). Consistent with the laying date not being significant, no optimum of laying date was found for the probability of hatching (Figure 7a). The probability of hatching was 0.73 on average.

#### 3.6.2 Hatchling to fledgling

This transition had a clear, supported optimum of survival according to laying date. The best model for this variable included laying date, size and age (see Supporting Information Table S5). Survival from hatchling stage to fledgling stage was lower for older (effect ± *SE* = −0.801 ± 0.258, *z* = −3.10, *p* = 0.00195) and younger (effect ± *SE* = −0.470 ± 0.105, *z* = 4.48, *p* = 7.38 × 10^−6^) females when compared to middle‐aged females (aged 2–6). It was positively associated with female tarsus size (slope ± *SE* = 0.155 ± 0.0722 mm^−1^, *z* = 2.149, *p* = 0.0316). The optimum of survival according to laying date declined from a maximum probability of survival of 0.82 to a minimum of 0.12 (Figure 7b). The optimum of survival was estimated at October 15th with a 95% credible interval between October 4th and October 24th. It is thus significantly different from the mode of laying date for this subset of the data (November 2nd). The probability of survival from hatchling to fledgling was 0.56 on average.

#### 3.6.3 Fledgling to recruit

The probability of survival and recruitment into the breeding population after fledgling did not significantly depend on any of the covariates tested in this study (see Supporting Information Table S6). It was low, at 0.22 on average.

#### 3.6.4 Egg to recruit

The compounded probability of surviving from the egg stage to recruitment had a clear optimum with the best model for this probability of survival including the effect of laying date and female age on the hatchling to fledgling transition. Survival was lower for offspring from younger (effect ± *SE* = −0.301 ± 0.129, *z* = −2.32, *p* = 0.0201) and older (effect ± *SE* = −1.08 ± 0.436, *z* = −2.47, *p* = 0.0134) females. The optimum of this probability of survival was estimated as October 3rd with a 95% credible interval between September 28th and October 12th (Figure 7d). It was thus significantly different from the mode of laying date for this subset of the data (November 1st). Total survival to breeding was overall very low with an average at 0.092.

## 4 DISCUSSION

Phenology is a key feature in adaptation to climate change for a broad spectrum of species (Parmesan & Yohe, 2003) and thus also an important aspect of the long‐term conservation of threatened species (Rosemartin et al., 2014; Wadgymar, Cumming, & Weis, 2015). To the best of our knowledge, this is the first study on a threatened species that has explored the relationship between a phenological trait, its genetic basis and fitness. Our results show that (a) there is an apparent discrepancy between the phenology of the hihi and its optimal value; (b) laying date is not significantly heritable, hence there is not sufficient genetic variation for the population to respond to natural selection; and (c), over the 16 years of this study, hihi phenology did not change towards earlier dates, although it is unclear whether it changed in the other direction or not. These results raise several questions and issues for both evolutionary and management perspectives, particularly because the population of Tiritiri Matangi continues to demonstrate strong population growth, despite a particularly large discrepancy between observed and optimal breeding times.

### 4.1 Discrepancy between laying date and its optimal value

We observed a very large difference between the optimum and mode of laying date (with an optimum almost a month earlier than the mode), with relatively few individuals that are actually breeding during the optimal period (see Figure 5). This gap results in an overall large selection differential of −11.24, which is much stronger than selection differentials estimated in other passerines (Gienapp et al., 2008; Van Noordwijk, McCleery, & Perrins, 1995; Visser, van Noordwijk, Tinbergen, & Lessells, 1998). This indicates a strong maladaptive phenology of the hihi population in Tiritiri Matangi Island, most likely imposing a burden in terms of population fitness, raising concern over the conservation status of this population. We found that phenology depended on a temperature cue (or, rather, a proxy of it) based on the average temperature 50 days prior to average start of breeding season. Yet, we found no significant connection between this cue and the optimum of laying date. This could be explained by a lack of power in our analysis or (not exclusively) by a weak relationship between this temperature cue and the optimum in Tiritiri Matangi. If this is the case, it would suggest that plasticity in response to this temperature cue is not efficient and adaptive enough to place the population close to the optimum. Data on more years is necessary to confirm this result. This is important for the hihi in the context of climate change as a negative relationship between increase in temperature and breeding success has been found, and projected regional climate change scenarios result in an overall decrease in the carrying capacity of the Tiritiri Matangi population (Chauvenet et al., 2013). However, should the temperature increase at Tiritiri Matangi, the observed phenotypic plasticity may become adaptive, as it would trigger earlier lay dates, possibly resolving the discrepancy with the optimum of laying date at the same time. This scenario cannot be tested here as the temperature cue did not increase over time during our study period, a period of time which overlaps with a “hiatus” in global climate change (Pachauri et al., 2014).

Despite the observed discrepancy between observed and optimal breeding times, the population of Tiritiri Matangi continues to demonstrate strong population growth. One hypothesis to explain this result is that recruitment is density‐dependent, with low reproductive output—due to the population breeding away from the optimum—compensated by high rates of fledgling recruitment in the absence of competition (Reed, Grøtan, Jenouvrier, Sæther, & Visser, 2013). However, no density‐dependent compensation has been observed in hihi (Armstrong & Ewen, 2013). Other compensatory mechanisms may be at play to explain the strong population growth, or there may be little cost associated with the discrepancy observed (Dunn & Møller, 2014; for example due to food supply being available ad libitum during all of the breeding season). This suggests that if some of the costs (large or small) associated with the discrepancy in lay date and suboptimal habitat were not in place, population growth would have an even more positive lambda than currently observed (Armstrong & Ewen, 2013).

### 4.2 Can individual quality explain the observed delay?

The difference between optimal and observed lay date could be explained by the fact that a part of the population is not fit enough to actively track this optimum, hence are obligatory late breeders, resulting in the evolution of an advanced laying date (Price, Kirkpatrick, & Arnold, 1988). This has been repeatedly found to be the case for fledgling success among passerines, where competition for resources or limitations in individual quality may mean that less fit individuals are unable to lay at the optimum (Van Noordwijk et al., 1995; Verhulst & Nilsson, 2008; Verhulst, van Balen, & Tinbergen, 1995). There are a number of lines of evidence working against this hypothesis in the case of the hihi. We found that the main factor related to individual quality explaining the differences in start of breeding was age, that is middle‐aged females tended to have the earliest start, as previously observed for many life‐history traits (Brekke et al., 2013; Chauvenet et al., 2013; Low, Pärt, & Forslund, 2007). However, estimating the optimum from only middle‐aged individuals returned essentially the same results as the inference using the general population, with an optimum still significantly earlier than the mode of laying date. Likewise, using only females surviving to the next year, as a proxy for high‐quality females, did not impact our results. The inability of even the seemingly fittest females to match the optimum suggests that direct competition between females is not the primary driver for the discrepancy in lay date. Differences in body condition may also contribute to individual differences in lay date. Unfortunately, female hihi body weight has not been consistently recorded over the period of study, with data only available for a small number of females (*n* = 36, Low, 2004, 2006). Regardless, the low repeatability of laying date suggests that there are very few consistently high‐quality females in the population across years. Finally, there is clear decline in fitness for females that are too early compared to the optimum (Figure 5), working against the hypothesis that “earliest is best.” Nevertheless, only an experimental approach (generally not possible in threatened species) can definitely disentangle the relative contributions of female quality and the effect of phenology to the observed pattern of fitness (Verhulst & Nilsson, 2008).

Another explanation for the difference between the optimum and mode of laying date would be a trade‐off between the reproductive output of a breeding event and survival to, or opportunity for, future breeding events. Again, this is unlikely to be the case in this system. Regarding opportunities for future breeding events within the same year, earliest breeding females were also the ones most likely to have a least one other clutch during the year. As for survival to future breeding events in consecutive years, the probability of female survival to consecutive years was not dependent on the phenology, and females that laid more clutches were more, not less, likely to survive (note that this is also the case when using survival according to surveys performed twice a year on the island, data not shown). Thus, early reproduction does not appear to come at a fitness cost for individual females.

### 4.3 Life‐history optimum and relationship to the ecology of the hihi

The fitness of a female's breeding event, as it was computed in this study, depended on two factors: the initial investment (number of eggs laid by the female) and the success of each individual investment (here separated into the survival at three developmental transitions from egg to hatchling, to fledgling, to recruit). The only significant impact of lay date on fitness that we found was during the transition from hatching to fledgling, with a relatively sharp and early optimum of laying date which increased survival. However, this relationship was strong enough to be a significant shaping factor of the overall probability of survival of young (i.e., we found a significant quadratic effect of laying date from egg to recruit). Before fledging, the parents are heavily dependent on the resources in their environment to provide for the nestlings. It has been shown, for example, that hihi adults drastically increase their consumption of invertebrates during this period (Castro et al., 1994), possibly to feed them to the juveniles (Rasch, 1985). Because these resources (invertebrates, but also fruit and flowers) will fluctuate throughout the breeding season, a reasonable explanation for our results is that laying eggs around mid‐October might coincide with a peak in specific resources for provisioning nestlings, around early November. However, in the context of this study, it is difficult to pinpoint the exact nature of these resources.

### 4.4 Lack of adaptive potential and phenotypic plasticity

The discrepancy between the optimum and the mode of laying date raises the question of the real ability of the population to face this challenge. Our results suggest that laying date is not significantly heritable, and hence, there is not sufficient genetic variation for the population to respond to the strong selection pressure through the means of natural selection. A difference in habitat quality between the remnant population on Hauturu‐o‐Toi and Tiritiri Matangi might partly explain a lack of adaptive potential in Tiritiri Matangi, if we assume a strong genotype‐by‐environment interaction that is masking the presence of additive genetic variance. Given that Tiritiri Matangi is in many respects more typical of the current state of New Zealand wild forests than Hauturu‐o‐Toi, especially in terms of forest maturity and complexity, it appears likely that in the majority of translocated hihi populations there is similarly no “exposed” additive genetic variation for selection to act on. Therefore, the majority of hihi populations appear likely to share low effective heritabilities for lay date and will be limited in their evolutionary response to selection. This effect would also be reflected in most endemic and endangered birds in New Zealand, who share a similar history of decline and reintroduction.

The lay date appeared to be responding to the temperature cue we analysed in this study, demonstrating the plasticity of phenology in the hihi. This is also confirmed by considering the Zealandia Eco‐sanctuary population (Karori, Wellington, New Zealand), which was predominantly founded from the Tiritiri Matangi population: Average female lay date is November 22nd (unpublished data), a difference of 20 days to females on Tiritiri Matangi, possibly reflecting colder conditions in this Southern location. However, we did not find a relationship between this temperature cue and the optimum of laying date, which suggests this temperature cue is not a very efficient predictor of the Tiritiri Matangi optimum. Environmental cues which may be present in the last remaining natural habitat Hauturu‐o‐Toi are probably absent or misleading in Tiritiri Matangi and therefore do not induce an adaptive response. Apart from the tested temperature cue, other cues for lay date are currently unknown and it is therefore challenging to identify the possible suite of cues that determine lay date, especially as strong evidence about the cue(s) would require experimental work, opportunity for which is limited for the hihi. Further, the forest of Tiritiri Matangi is still regenerating and climate is predicted to also change, which will likely result in a modification of the cues, the optimum and the mode of laying date in the future. Whether these changes over time will resolve or increase the discrepancy with the optimum is hence unknown.

### 4.5 Conservation implications and future management

The hihi conservation programme, like many other New Zealand conservation programmes, is limited by sites that can deal with the main threats to its survival—mammalian predators and loss of pristine habitat. Mammalian predator control has been achieved with the use of island sites or large‐scale fencing of on‐shore sanctuaries. However, finding large enough forested areas that contain restored habitat (as most pristine, mature forests have been cleared) but could sustain a hihi population with ongoing management remains a challenge. Despite this, the hihi programme has been successful at establishing new, growing populations at a range of sites on off‐shore and mainland islands alike. But the threat of climate change remains a constant, and populations at these new sites limited to suboptimal, immature habitat are likely to be more susceptible to its effects, as they are already at a disadvantage from displaying a discrepancy with current environmental conditions, potentially limiting their long‐term viability. The lack of adaptive potential and adaptive plasticity in this species may be one of the reasons it requires intense management to maintain the reintroduced populations. However, this level of intervention is not unusual in highly threatened species, and the established populations continue to grow and flourish in relatively varied habitats across the North Island. Our findings, along with previous research (Chauvenet et al., 2013), support the emphasis on assisted colonization to areas outside the hihi natural range, as climate changes and these areas potentially become more suitable over the coming decades. However, given that plasticity is not perfectly adaptive and given the challenges associated with identifying the environmental cues, it is difficult to make predictions about the suitability of these new sites to resolve the discrepancy between observed and optimal laying date.

As the number of threatened species increases because of anthropogenic action, so do the number and types of human intervention to prevent extinction. One of the most commonly used tools for the management of threatened species is reintroduction (Ewen, Armstrong, Parker, & Seddon, 2011), currently being used in hundreds of conservation programmes globally, across most taxa (Soorae, 2016). In the process of reintroduction, threatened populations undergo genetic bottlenecks, as a consequence of sampling a small number of individuals to found new populations. The repercussions of bottlenecks are well established: loss of genetic diversity and, consequently, loss of adaptive potential (Willi, Buskirk, & Hoffmann, 2006). But in the face of extinction, this trade‐off may be the only one or one of very few alternative/s. Conservation programmes globally are also limited by large‐scale habitat loss, leaving feasible sites for reintroduction at best modified but more commonly suboptimal or even nonexistent. Finding appropriate reintroduction sites therefore remains a huge management challenge, particularly as for most species there is no information on optimal habitat (Armstrong, Castro, & Griffiths, 2007). Here, we show that for threatened species with low genetic diversity in the face of climate change, surviving in suboptimal habitat can add another level of complexity. Populations in suitable (for conservation purpose, e.g., with a positive population growth, as is the studied population here), but suboptimal (from an evolutionary perspective), habitat are likely to be more susceptible to the effects of climate change as they are already at a disadvantage from displaying a discrepancy with current environmental conditions, potentially further limiting their long‐term viability.

Our findings precisely quantify adaptive potential and plasticity in a trait vital to population fitness in a closed population of a threatened species. Ongoing debate has centred on the relative importance of these two processes to how species may overcome the effects of climate change. We are the first to show empirical evidence towards how an already threatened species may cope (or not), but more evidence is sorely needed on a larger number of traits, populations and species to enable us to assess more accurately its effects and test genetic management alternatives more widely.

## CONFLICT OF INTEREST

None declared.

## AUTHORS CONTRIBUTION

AWS and PB conceived of the study. JGE and PB supervised and coordinated the collection of the data. PB developed the microsatellite data set, supervised the genotyping and performed the pedigree reconstruction. PdV designed and conducted the analysis of the data, with advice from all other authors. PdV wrote the paper, with input from all other authors.

## ETHICAL STATEMENT

Permissions to conduct research and collect blood samples for parentage analysis on Tiritiri Matangi were granted by the New Zealand Department of Conservation, permit numbers 36186‐FAU, 15073‐RES, 24128‐FAU, 13939‐RES and 44300‐FAU.

## Data Availability

The data, model and simulation code are available online on the Dryad database (https://doi.org/10.5061/dryad.0h2br0d).

## References

[eva12727-bib-0001] Akaike, H. (1981). Likelihood of a model and information criteria. Journal of Econometrics, 16(1), 3–14.

[eva12727-bib-0002] Armstrong, D. P. , Castro, I. , & Griffiths, R. (2007). Using adaptive management to determine requirements of re‐introduced populations: The case of the New Zealand hihi. Journal of Applied Ecology, 44(5), 953–962. 10.1111/j.1365-2664.2007.01320.x

[eva12727-bib-0003] Armstrong, D. P. , & Ewen, J. G. (2013). Consistency, continuity and creativity: Long‐term studies of population dynamics on Tiritiri Matangi Island. New Zealand Journal of Ecology, 37(3), 288.

[eva12727-bib-0004] Bates, D. , Mächler, M. , Bolker, B. , & Walker, S. (2015). Fitting linear mixed‐effects models using lme4. Journal of Statistical Software, 67(1), 48.

[eva12727-bib-0005] Bellard, C. , Bertelsmeier, C. , Leadley, P. , Thuiller, W. , & Courchamp, F. (2012). Impacts of climate change on the future of biodiversity: Biodiversity and climate change. Ecology Letters, 15(4), 365–377. 10.1111/j.1461-0248.2011.01736.x 22257223 PMC3880584

[eva12727-bib-0006] Both, C. , Artemyev, A. V. , Blaauw, B. , Cowie, R. J. , Dekhuijzen, A. J. , Eeva, T. , … Visser, M. E. (2004). Large–scale geographical variation confirms that climate change causes birds to lay earlier. Proceedings of the Royal Society of London B: Biological Sciences, 271(1549), 1657–1662. 10.1098/rspb.2004.2770 PMC1691776 15306284

[eva12727-bib-0007] Brekke, P. , Cassey, P. , Ariani, C. , & Ewen, J. G. (2013). Evolution of extreme‐mating behaviour: Patterns of extrapair paternity in a species with forced extrapair copulation. Behavioral Ecology and Sociobiology, 67(6), 963–972. 10.1007/s00265-013-1522-9

[eva12727-bib-0008] Brekke, P. , Dawson, D. A. , Horsburgh, G. J. , & Ewen, J. G. (2009). Characterization of microsatellite loci in the hihi *Notiomystis cincta* (Notiomystidae, Aves). Molecular Ecology Resources, 9(4), 1255–1258. 21564894 10.1111/j.1755-0998.2009.02626.x

[eva12727-bib-0009] Brekke, P. , Ewen, J. G. , Clucas, G. , & Santure, A. W. (2015). Determinants of male floating behaviour and floater reproduction in a threatened population of the hihi (*Notiomystis cincta*). Evolutionary Applications, 8(8), 796–806. 10.1111/eva.12287 26366197 PMC4561569

[eva12727-bib-0010] Brekke, P. , Wang, J. , Bennett, P. M. , Cassey, P. , Dawson, D. A. , Horsburgh, G. J. , & Ewen, J. G. (2012). Postcopulatory mechanisms of inbreeding avoidance in the island endemic hihi (*Notiomystis cincta*). Behavioral Ecology, 23(2), 278–284. 10.1093/beheco/arr183

[eva12727-bib-0011] Brown, C. R. , & Brown, M. B. (1999). Fitness components associated with laying date in the cliff swallow. Condor, 101(2), 230–245. 10.2307/1369986

[eva12727-bib-0012] Burnham, K. P. , & Anderson, D. R. (2002). Model selection and multimodel inference: A practical informationtheoretic approach. Berlin, Germany: Springer.

[eva12727-bib-0013] Caro, S. P. , Schaper, S. V. , Hut, R. A. , Ball, G. F. , & Visser, M. E. (2013). The case of the missing mechanism: How does temperature influence seasonal timing in endotherms? PLOS Biology, 11(4), e1001517 10.1371/journal.pbio.1001517 23565055 PMC3614498

[eva12727-bib-0014] Castro, I. , Minot, E. O. , & Alley, J. C. (1994). Feeding and breeding behaviour of hihi or stitchbirds *Notiomystis cincta* recently transferred to Kapiti Island, New Zealand, and possible management alternatives In Serena M. (Ed.), Reintroduction biology of Australian and New Zealand fauna (pp. 121–128). Chipping Norton, NSW: Surrey Beatty & Sons Pty Ltd.

[eva12727-bib-0015] Charmantier, A. , McCleery, R. H. , Cole, L. R. , Perrins, C. , Kruuk, L. E. B. , & Sheldon, B. C. (2008). Adaptive phenotypic plasticity in response to climate change in a wild bird population. Science, 320(5877), 800–803. 10.1126/science.1157174 18467590

[eva12727-bib-0016] Chauvenet, A. L. M. , Ewen, J. G. , Armstrong, D. , & Pettorelli, N. (2013). Saving the hihi under climate change: A case for assisted colonization. Journal of Applied Ecology, 50(6), 1330–1340. 10.1111/1365-2664.12150

[eva12727-bib-0017] Chevin, L.‐M. , & Lande, R. (2015). Evolution of environmental cues for phenotypic plasticity. Evolution, 69(10), 2767–2775. 10.1111/evo.12755 26292649

[eva12727-bib-0018] Chevin, L.‐M. , Visser, M. E. , & Tufto, J. (2015). Estimating the variation, autocorrelation, and environmental sensitivity of phenotypic selection. Evolution, 69(9), 2319–2332. 10.1111/evo.12741 26227394

[eva12727-bib-0019] Chuine, I. (2010). Why does phenology drive species distribution? Philosophical Transactions of the Royal Society of London B: Biological Sciences, 365(1555), 3149–3160. 10.1098/rstb.2010.0142 20819809 PMC2981946

[eva12727-bib-0020] Crick, H. Q. P. , Dudley, C. , Glue, D. E. , & Thomson, D. L. (1997). UK birds are laying eggs earlier. Nature, 388(6642), 526 10.1038/41453

[eva12727-bib-0021] Dawson, D. A. , Åkesson, M. , Burke, T. , Pemberton, J. M. , Slate, J. , & Hansson, B. (2007). Gene order and recombination rate in homologous chromosome regions of the chicken and a passerine bird. Molecular Biology and Evolution, 24(7), 1537–1552. 10.1093/molbev/msm071 17434902

[eva12727-bib-0022] de Villemereuil, P. , Morrissey, M. B. , Nakagawa, S. , & Schielzeth, H. (2018). Fixed effect variance and the estimation of repeatabilities and heritabilities: Issues and solutions. Journal of Evolutionary Biology, 31(4), 621–632. 10.1111/jeb.13232 29285829

[eva12727-bib-0023] Dunn, P. O. , & Møller, A. P. (2014). Changes in breeding phenology and population size of birds. Journal of Animal Ecology, 83(3), 729–739. 10.1111/1365-2656.12162 24117440

[eva12727-bib-0024] Ewen, J. G. , & Armstrong, D. P. (2000). Male provisioning is negatively correlated with attempted extrapair copulation frequency in the stitchbird (or hihi). Animal Behaviour, 60(4), 429–433. 10.1006/anbe.2000.1485 11032645

[eva12727-bib-0025] Ewen, J. G. , Armstrong, D. P. , Parker, K. A. , & Seddon, P. J. (Eds.) (2011). Reintroduction biology: Integrating science and management. Chichester, UK: Blackwell Publishing Ltd, John Wiley & Sons.

[eva12727-bib-0026] Gelman, A. , Meng, X. L. , & Stern, H. (1996). Posterior predictive assessment of model fitness via realized discrepancies. Statistica Sinica, 6, 733–759.

[eva12727-bib-0027] Gelman, A. , & Rubin, D. B. (1992). Inference from iterative simulation using multiple sequences. Statistical Science, 7(4), 457–472.

[eva12727-bib-0028] Ghalambor, C. K. , McKay, J. K. , Carroll, S. P. , & Reznick, D. N. (2007). Adaptive versus non‐adaptive phenotypic plasticity and the potential for contemporary adaptation in new environments. Functional Ecology, 21(3), 394–407. 10.1111/j.1365-2435.2007.01283.x

[eva12727-bib-0029] Gienapp, P. , Postma, E. , & Visser, M. E. (2006). Why breeding time has not responded to selection for earlier breeding in a songbird population. Evolution, 60(11), 2381–2388. 10.1111/j.0014-3820.2006.tb01872.x 17236428

[eva12727-bib-0030] Gienapp, P. , Teplitsky, C. , Alho, J. S. , Mills, J. A. , & Merilä, J. (2008). Climate change and evolution: Disentangling environmental and genetic responses. Molecular Ecology, 17(1), 167–178. 10.1111/j.1365-294X.2007.03413.x 18173499

[eva12727-bib-0031] Hadfield, J. D. (2010). MCMC methods for multi‐response generalized linear mixed models: The MCMCglmm R package. Journal of Statistical Software, 33(2), 1–22.20808728

[eva12727-bib-0032] Heidelberger, P. , & Welch, P. D. (1981). A spectral method for confidence interval generation and run length control in simulations. Communications of the ACM, 24(4), 233–245. 10.1145/358598.358630

[eva12727-bib-0033] Lande, R. , & Arnold, S. J. (1983). The measurement of selection on correlated characters. Evolution, 37(6), 1210–1226. 10.2307/2408842 28556011

[eva12727-bib-0034] Low, M. (2004). Female weight predicts the timing of forced copulation attempts in stitchbirds, *Notiomystis cincta* . Animal Behaviour, 68(3), 637–644. 10.1016/j.anbehav.2004.01.006

[eva12727-bib-0035] Low, M. (2006). Sex, age and season influence morphometrics in the New Zealand Stitchbird (or Hihi; *Notiomystis cincta*). Emu, 106(4), 297 10.1071/MU06003

[eva12727-bib-0036] Low, M. , Joy, M. K. , & Makan, T. (2006). Using regression trees to predict patterns of male provisioning in the stitchbird (hihi). Animal Behaviour, 71(5), 1057–1068. 10.1016/j.anbehav.2005.07.021

[eva12727-bib-0037] Low, M. , & Pärt, T. (2009). Patterns of mortality for each life‐history stage in a population of the endangered New Zealand stitchbird. Journal of Animal Ecology, 78(4), 761–771. 10.1111/j.1365-2656.2009.01543.x 19302320

[eva12727-bib-0038] Low, M. , Pärt, T. , & Forslund, P. (2007). Age‐specific variation in reproduction is largely explained by the timing of territory establishment in the New Zealand stitchbird *Notiomystis cincta* . Journal of Animal Ecology, 76(3), 459–470. 10.1111/j.1365-2656.2007.01234.x 17439463

[eva12727-bib-0039] Makan, T. , Castro, I. , Robertson, A. W. , Joy, M. K. , & Low, M. (2014). Habitat complexity and management intensity positively influence fledging success in the endangered hihi (*Notiomystis cincta*). New Zealand Journal of Ecology, 38(1), 53–63.

[eva12727-bib-0040] McLean, N. , Lawson, C. R. , Leech, D. I. , & van de Pol, M. (2016). Predicting when climate‐driven phenotypic change affects population dynamics. Ecology Letters, 19(6), 595–608. 10.1111/ele.12599 27062059

[eva12727-bib-0041] Merilä, J. , & Sheldon, B. C. (2000). Lifetime reproductive success and heritability in nature. American Naturalist, 155(3), 301–310. 10.1086/303330PMID: 10718727. 10718727

[eva12727-bib-0042] Mittell, E. A. , Nakagawa, S. , & Hadfield, J. D. (2015). Are molecular markers useful predictors of adaptive potential? Ecology Letters, 18(8), 772–778. 10.1111/ele.12454 25989024

[eva12727-bib-0043] Pachauri, R. K. , Allen, M. R. , Barros, V. R. , Broome, J. , Cramer, W. , Christ, R. , … Dasgupta, P. (2014). Climate change 2014: Synthesis report. Contribution of Working Groups I, II and III to the fifth assessment report of the Intergovernmental Panel on Climate Change. Geneva, Switzerland: Intergovernmental Panel on Climate Change.

[eva12727-bib-0044] Parmesan, C. , & Yohe, G. (2003). A globally coherent fingerprint of climate change impacts across natural systems. Nature, 421(6918), 37–42. 10.1038/nature01286 12511946

[eva12727-bib-0045] Phillimore, A. B. , Leech, D. I. , Pearce‐Higgins, J. W. , & Hadfield, J. D. (2016). Passerines may be sufficiently plastic to track temperature‐mediated shifts in optimum lay date. Global Change Biology, 22(10), 3259–3272. 10.1111/gcb.13302 27173755

[eva12727-bib-0046] Plummer, M. (2003). JAGS: A program for analysis of Bayesian graphical models using Gibbs sampling. Proceedings of the 3rd International Workshop on Distributed Statistical Computing, March (pp. 20–22).

[eva12727-bib-0047] Plummer, M. , Best, N. , Cowles, K. , & Vines, K. (2006). CODA: Convergence diagnosis and output analysis for MCMC. R News, 6(1), 7–11.

[eva12727-bib-0048] Price, T. , Kirkpatrick, M. , & Arnold, S. (1988). Directional selection and the evolution of breeding date in birds. Science, 240(4853), 798–799. 10.1126/science.3363360 3363360

[eva12727-bib-0049] R Core Team . (2017). R: A language and environment for statistical computing. Vienna, Austria: R Foundation for Statistical Computing.

[eva12727-bib-0050] Rasch, G. (1985). The ecology of cavity nesting in the stitchbird (*Notiomystis cincta*). New Zealand Journal of Zoology, 12(4), 637–642. 10.1080/03014223.1985.10428313

[eva12727-bib-0051] Reed, T. E. , Grøtan, V. , Jenouvrier, S. , Sæther, B.‐E. , & Visser, M. E. (2013). Population growth in a wild bird is buffered against phenological mismatch. Science, 340(6131), 488–491. 10.1126/science.1232870 23620055

[eva12727-bib-0052] Robertson, A. (1966). A mathematical model of the culling process in dairy cattle. Animal Science, 8(01), 95–108. 10.1017/S0003356100037752

[eva12727-bib-0053] Robertson, A. (1968). Population biology and evolution (pp. 5–16). New York, NY: Syracuse University Press.

[eva12727-bib-0054] Rosemartin, A. H. , Crimmins, T. M. , Enquist, C. A. F. , Gerst, K. L. , Kellermann, J. L. , Posthumus, E. E. , … Weltzin, J. F. (2014). Organizing phenological data resources to inform natural resource conservation. Biological Conservation, 173, 90–97. 10.1016/j.biocon.2013.07.003

[eva12727-bib-0055] Rubin, D. B. (1984). Bayesianly justifiable and relevant frequency calculations for the applies statistician. Annals of Statistics, 12(4), 1151–1172.

[eva12727-bib-0056] Sheldon, B. C. , Kruuk, L. E. B. , & Merilä, J. (2003). Natural selection and inheritance of breeding time and clutch size in the collared flycatcher. Evolution, 57(2), 406–420. 10.1111/j.0014-3820.2003.tb00274.x 12683536

[eva12727-bib-0057] Soorae, P. S. (2016). Global re‐introduction perspectives: 2016. Case‐studies from around the globe. Gland, Switzerland: IUCN/SSC Re‐introduction Specialist Group & Environment Agency.

[eva12727-bib-0058] Teplitsky, C. , Mills, J. A. , Yarrall, J. W. , & Merilä, J. (2010). Indirect genetic effects in a sex‐limited trait: The case of breeding time in red‐billed gulls. Journal of Evolutionary Biology, 23(5), 935–944. 10.1111/j.1420-9101.2010.01959.x 20345824

[eva12727-bib-0059] Van Noordwijk, A. , McCleery, R. , & Perrins, C. (1995). Selection for the timing of great tit breeding in relation to caterpillar growth and temperature. Journal of Animal Ecology, 64(4), 451–458. 10.2307/5648

[eva12727-bib-0060] Verhulst, S. , & Nilsson, J.‐Å. (2008). The timing of birds’ breeding seasons: A review of experiments that manipulated timing of breeding. Philosophical Transactions of the Royal Society of London B: Biological Sciences, 363(1490), 399–410. 10.1098/rstb.2007.2146 17666390 PMC2606757

[eva12727-bib-0061] Verhulst, S. , van Balen, J. H. , & Tinbergen, J. M. (1995). Seasonal decline in reproductive success of the great tit: Variation in time or quality? Ecology, 76(8), 2392–2403. 10.2307/2265815

[eva12727-bib-0062] Visser, M. E. , Both, C. , & Lambrechts, M. M. (2004). Global climate change leads to mistimed Avian reproduction In B. A. I. E. Research (Ed.), Birds and climate change (vol. 35, pp. 89–110). Cambridge, MA: Academic Press.

[eva12727-bib-0063] Visser, M. E. , van Noordwijk, A. J. , Tinbergen, J. M. , & Lessells, C. M. (1998). Warmer springs lead to mistimed reproduction in great tits (*Parus major*). Proceedings of the Royal Society of London B: Biological Sciences, 265(1408), 1867–1870. 10.1098/rspb.1998.0514

[eva12727-bib-0064] Wadgymar, S. M. , Cumming, M. N. , & Weis, A. E. (2015). The success of assisted colonization and assisted gene flow depends on phenology. Global Change Biology, 21(10), 3786–3799. 10.1111/gcb.12988 26033188

[eva12727-bib-0065] Wang, J. (2004). Sibship reconstruction from genetic data with typing errors. Genetics, 166(4), 1963–1979. 10.1534/genetics.166.4.1963 15126412 PMC1470831

[eva12727-bib-0066] Wang, J. (2012). Computationally efficient sibship and parentage assignment from multilocus marker data. Genetics, 191(1), 183–194. 10.1534/genetics.111.138149 22367033 PMC3338259

[eva12727-bib-0067] Wang, J. , & Santure, A. W. (2009). Parentage and sibship inference from multilocus genotype data under polygamy. Genetics, 181(4), 1579–1594. 10.1534/genetics.108.100214 19221199 PMC2666522

[eva12727-bib-0068] Wickham, H. (2009). Ggplot2: Elegant graphics for data analysis. New York, NY: Springer‐Verlag.

[eva12727-bib-0069] Willi, Y. , Buskirk, J. V. , & Hoffmann, A. A. (2006). Limits to the adaptive potential of small populations. Annual Review of Ecology, Evolution, and Systematics, 37(1), 433–458. 10.1146/annurev.ecolsys.37.091305.110145

