# Supplementary material for: Can threatened species adapt in a restored habitat? No expected evolutionary response in lay date for the New Zealand hihi
Source: Evol Appl. 2018 Dec 12;12(3):482–97. doi: 10.1111/eva.12727 (PMC6383709; doi:10.1111/eva.12727)
Supplement: Supplementary file 1 [file EVA-12-482-s001.pdf]

Supplementary Information for "Can threatened species adapt in restored habitat? No expected evolutionary response in lay date for the New Zealand hihi" (Evolutionary Applications)

Pierre de Villemereuil, Alexis Rutschmann, John G. Ewen, Anna W. Santure & Patricia Brekke

Model selection: start of breeding season

Table S1: Selection of covariates to model start of breeding season. All the different models for age are presented (linear model, quadratic, broken lines with 1, 3 or all breaks). Because inbreeding requires a strong subsampling of the data, the AIC were re-computed on this smaller dataset.

Fixed effect	AIC	ΔAIC
General dataset		
**Age (3 breaks) + Size**	5630.5	0
Age (all breaks) + Size	5633.6	3.1
Age (1break) + Size	5638.2	7.7
Age (quadratic) + Size	5643.9	13.4
Age (linear) + Size	5741.2	110.7
Size	5759.6	129.1
None	5762.9	132.4
Subset with available inbreeding data		
**Age (3 breaks) + Size**	2555.3	0
Age (3 breaks) + Size + Inbreeding	2557.2	1.9
Inbreeding	2638.1	82.8
None	2638.4	83.1

Model selection: probability of reclutch

Table S2: Selection of covariates to model probability of reclutch. The different models for age tested are: continuous, First year Vs. others, Young (1)/Middle (2-6)/Old(>6). Because including inbreeding requires a strong subsampling of the data, the AIC were re-computed on this smaller dataset.

Fixed effect	AIC	ΔAIC
General dataset		
**Start + Age (First/Non First)**	823.0	0
Start + Age (Young/Middle/Old)	826.9	3.9
Start + Age (linear)	839.2	16.2
Start	847.7	24.7
None	1047.3	224.3
Subset with available inbreeding data		
**Start + Age (First/Non First)**	361.1	0
Start + Age (First/Non First) + Inbreeding	363.0	1.94
None	419.2	58.1
Inbreeding	420.6	59.5

Model selection and graph: survival of female to the next year

Table S3: Selection of covariates to model survival of the female to the next year. The different models for age tested are: continuous, First year Vs. others, Young (1)/Middle (2-6)/Old(>6). Because including inbreeding requires a strong subsampling of the data, the AIC were re-computed on this smaller dataset.

Fixed effect	AIC	ΔAIC
General dataset		
**Age + Nb. Clutch**	1013.5	0
Age (Young/Middle/Old)	1032.3	18.8
Nb. Clutch	1043.2	29.7
Age (First/Non First) + Nb. Clutch	1045.1	31.6
Start (quadratic)	1056.0	42.5
None	1056.2	42.7
Size	1057.4	43.9
Subset with available inbreeding data		
**Age + Nb. Clutch**	431.6	0
Age + Nb. Clutch + Inbreeding	432.2	0.6
None	445.8	14.2
Inbreeding	447.3	15.7

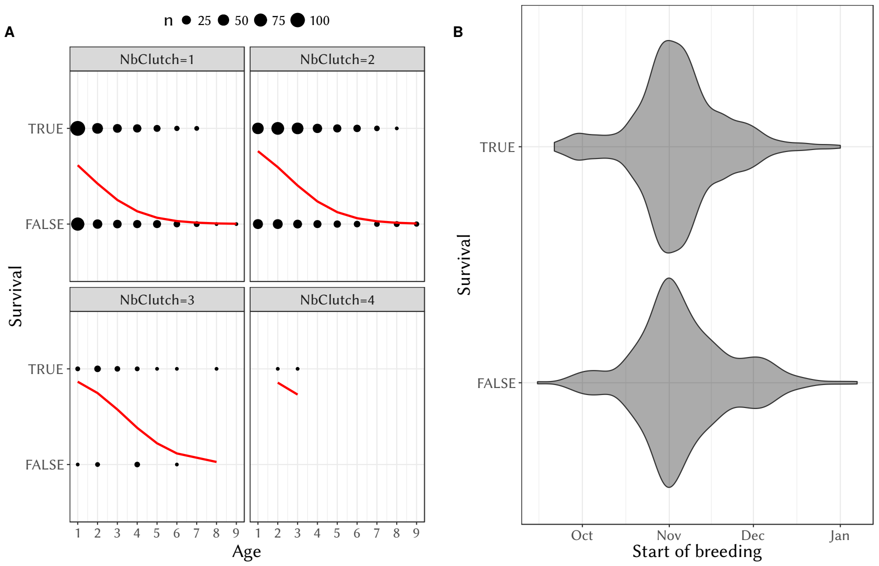

Figure S1: Survival of breeding females to the next year according to: A, their age and the number of clutch laid during the breeding season; and B, their start date of breeding (first laying date). The red lines depict the predicted values (plot A only, as start of breeding is not significant).

Power analysis

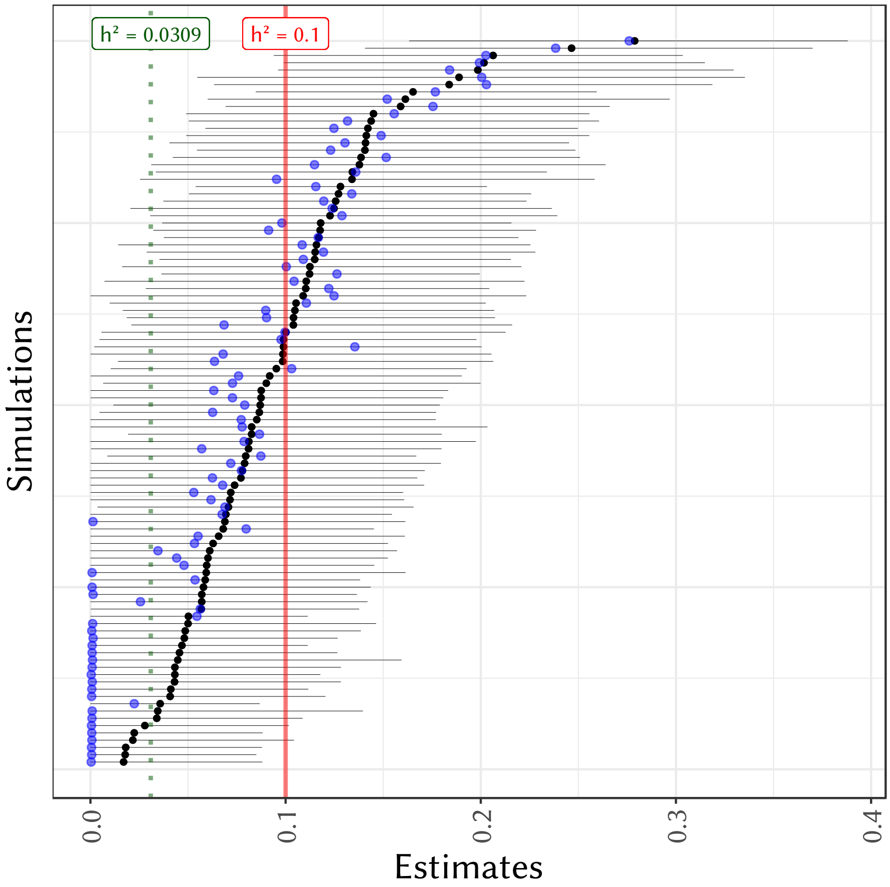

Figure S2: Posterior mode (light blue point), median (black point) and 95% credible interval (black line) for all 100 replicates of the power analysis. The light red line is the true heritability and dotted green line is the estimated posterior mode in our study.

Posterior predictive check

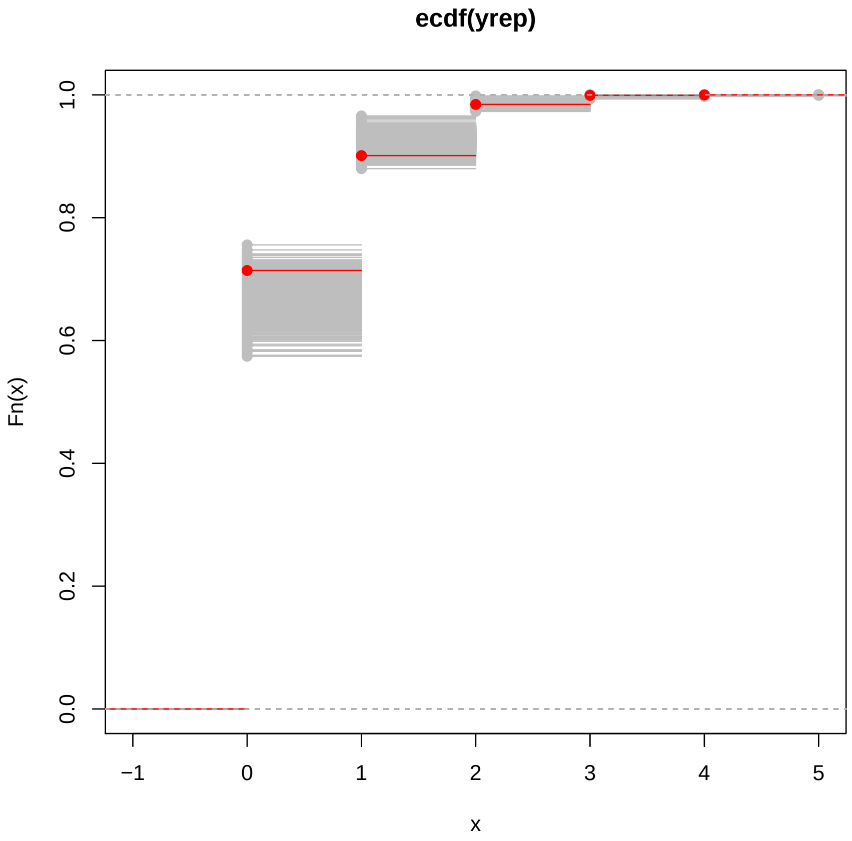

Figure S3: Posterior predictive check of the model to estimate the optimum of fitness. In grey: empirical cumulative distribution function (ECDF) of 1000 date replicates based on the model estimates and their posterior distribution. In red: ECDF of the observed data. We see that there is a slight enrichment in zero in the data, but not aberrant compared to the inferred model.

Optimum of laying date for females of "best quality"

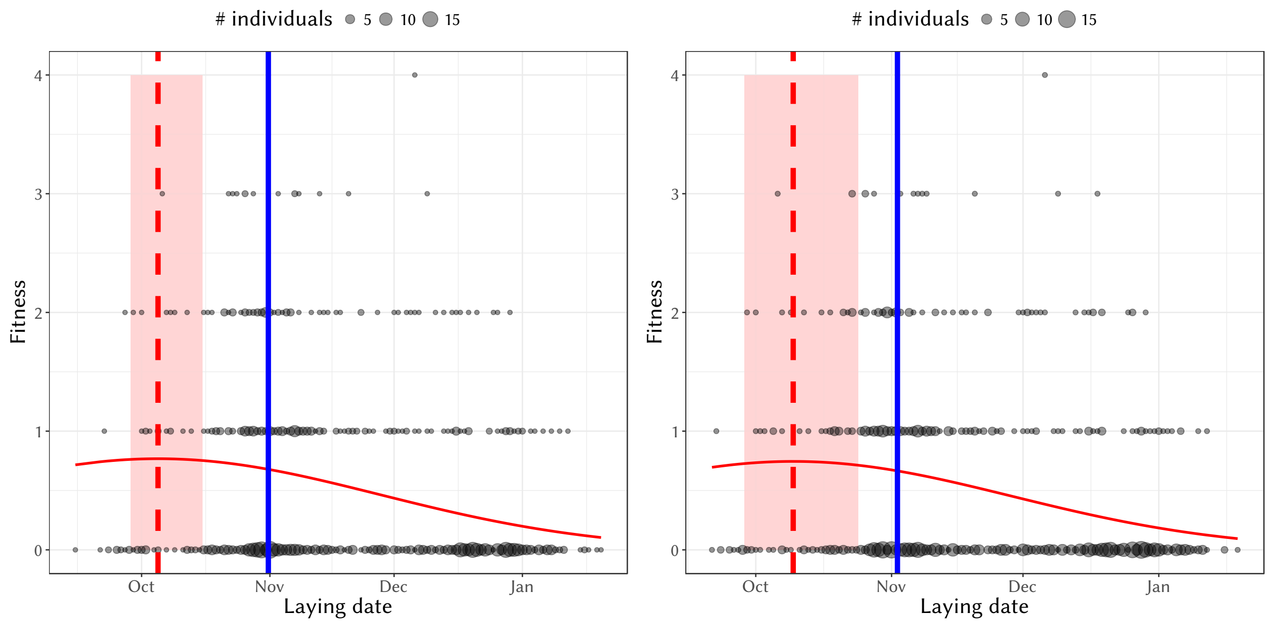

Figure S4: Fitness against laying date for mature females (left, age 2 to 6) and females surviving to consecutive years (right). Circle sizes are proportional to the number of individuals sharing the same fitness value and laying date. The red curve is the fitted model, vertical red dashed line is the optimum and the light red area depicts the 95% credible interval of the optimum. The vertical solid blue line is the mode of laying date. Fitness is defined as the number of offspring recruited as breeders in the following generations.

Optimum of start of breeding season

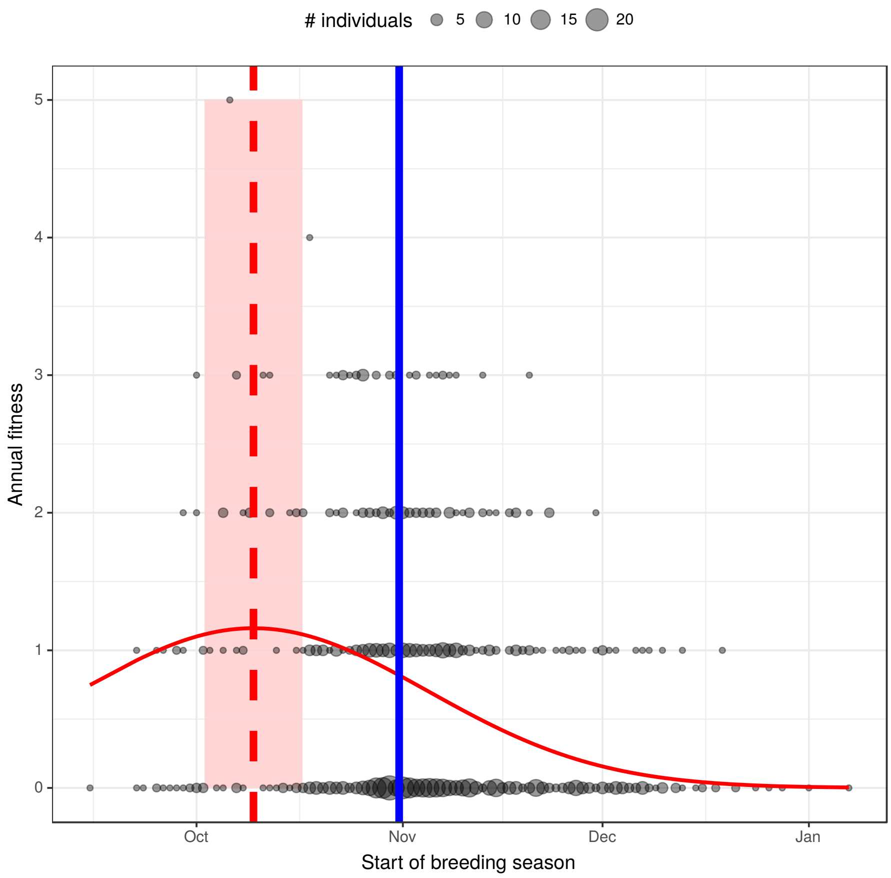

Figure S5: Annual fitness (cumulated number of fledglings) against the start of breeding date (date of the first clutch). Circle sizes are proportional to the number of individuals sharing the same fitness value and laying date. The red curve is the fitted model, vertical red dashed line is the optimum and the light red area depicts the 95% credible interval of the optimum. The vertical solid blue line is the mode of laying date. Fitness is defined as the number of offspring recruited as breeders in the following generations.

Model selection: survival between juvenile stages

From egg to hatchling

Table S4: Selection of covariates to model survival from egg to hatchling (probability of hatching). To keep the number of models tested low, only one model for age was tested: Young (1)/Middle (2-6)/Old(>6). Laying date (LD) was fitted using a quadratic effect. Because including inbreeding requires a strong subsampling of the data, the AIC were re-computed on this smaller dataset.

**Fixed effect**	**AIC**	**ΔAIC**
General dataset		
LD (quad) + Age (Young/Middle/Old) + Clutch	3318.6	0
**Age (Young/Middle/Old) + Clutch**	3318.7	0.1
Age (Young/Middle/Old)	3323.3	4.7
Clutch	3331.4	12.8
LD (quad)	3331.8	13.2
None	3334.6	16
Size	3336.3	17.7
Subset with available inbreeding data		
**Age (Young/Middle/Old) + Clutch**	1308.1	0
Age (Young/Middle/Old) + Clutch + Inbreeding	1309.1	1
None	1311.5	3.4
Inbreeding	1313	4.9

From hatchling to fledgling

Table S5: Selection of covariates to model survival from hatchling to fledgling. To keep the number of models tested low, only one model for age was tested: Young (1)/Middle (2-6)/Old(>6). Laying date (LD) was fitted using a quadratic effect. Because including inbreeding requires a strong subsampling of the data, the AIC were re-computed on this smaller dataset.

**Fixed effect**	**AIC**	**ΔAIC**
General dataset		
**LD (quad) + Size + Age (Young/Middle/Old)**	2907.7	0
LD (quad) + Size + Age (Young/Middle/Old) + Clutch	2908.6	0.9
LD (quad) + Age	2910.3	2.6
LD (quad) + Age (Young/Middle/Old) + Clutch	2911.2	3.5
LD (quad) + Size + Clutch	2927.7	20
LD (quad) + Size	2932	24.3
LD (quad)	2934.1	26.4
Size + Age (Young/Middle/Old) + Clutch	3067.4	159.7
Clutch	3135.3	227.6
Age (Young/Middle/Old) + Size	3534.6	626.9
Age (Young/Middle/Old)	3539.1	631.4
Size	3563.3	655.6
None	3567.3	659.6
Subset with available inbreeding data		
LD (quad) + Size + Age (Young/Middle/Old) + Inbreeding	1193.7	0
**LD (quad) + Size + Age (Young/Middle/Old)**	1194.6	0.9
Inbreeding	1472.7	279
None	1477.8	284.1

From fledgling to recruit

Table S6: Selection of covariates to model survival from fledgling to recruit. To keep the number of models tested low, only one model for age was tested: Young (1)/Middle (2-6)/Old(>6). Laying date (LD) was fitted using a quadratic effect. Because including inbreeding requires a strong subsampling of the data, the AIC were re-computed on this smaller dataset.

**Fixed effect**	**AIC**	**ΔAIC**
General dataset		
**None**	1356.8	0
Size	1357.7	0.9
Clutch	1358.5	1.7
Age (Young/Middle/Old)	1359.5	2.7
LD (quad)	1359.8	3
Subset with available inbreeding data		
**None**	498.9	0
Inbreeding	500.8	1.9

From egg to recruit

Table S7: Selection of covariates to model survival from egg to recruit. To keep the number of models tested low, only one model for age was tested: Young (1)/Middle (2-6)/Old(>6). Laying date (LD) was fitted using a quadratic effect. Because including inbreeding requires a strong subsampling of the data, the AIC were re-computed on this smaller dataset.

**Fixed effect**	**AIC**	**ΔAIC**
General dataset		
**LD (quad) + Age (Young/Middle/Old)**	1747.2	0
LD (quad) + Age (Young/Middle/Old) + Clutch	1749.1	1.9
LD (quad)	1755.1	7.9
LD (quad) + Clutch	1756.9	9.7
Age (Young/Middle/Old) + Clutch	1764.8	17.6
Clutch	1778.5	31.3
Age (Young/Middle/Old)	1818.3	71.1
Size	1827.1	79.9
None	1827.2	80
Subset with available inbreeding data		
**LD (quad) + Age (Young/Middle/Old)**	649	0
LD (quad) + Age (Young/Middle/Old) + Inbreeding	650.7	1.7
None	677.1	28.1
Inbreeding	678.2	29.2
